# Heterostructured SnO_2_-SnS_2_@C Embedded in Nitrogen-Doped Graphene as a Robust Anode Material for Lithium-Ion Batteries

**DOI:** 10.3389/fchem.2019.00339

**Published:** 2019-05-14

**Authors:** Hui Li, Bao Zhang, Xu Wang, Jie Zhang, Tianhui An, Zhiying Ding, Wanjing Yu, Hui Tong

**Affiliations:** ^1^School of Metallurgy and Environment, Central South University, Changsha, China; ^2^School of Chemistry and Chemical Engineering, Central South University, Changsha, China

**Keywords:** SnO_2_-SnS_2_, heterostructure, nitrogen-doped graphene, nanoparticle, anode

## Abstract

Tin-based anode materials with high capacity attract wide attention of researchers and become a strong competitor for the next generation of lithium-ion battery anode materials. However, the poor electrical conductivity and severe volume expansion retard the commercialization of tin-based anode materials. Here, SnO_2_-SnS_2_@C nanoparticles with heterostructure embedded in a carbon matrix of nitrogen-doped graphene (SnO_2_-SnS_2_@C/NG) is ingeniously designed in this work. The composite was synthesized by a two-step method. Firstly, the SnO_2_@C/rGO with a nano-layer structure was synthesized by hydrothermal method as the precursor, and then the SnO_2_-SnS_2_@C/NG composite was obtained by further vulcanizing the above precursor. It should be noted that a carbon matrix with nitrogen-doped graphene can inhibit the volume expansion of SnO_2_-SnS_2_ nanoparticles and promote the transport of lithium ions during continuous cycling. Benefiting from the synergistic effect between nanoparticles and carbon matrix with nitrogen-doped graphene, the heterostructured SnO_2_-SnS_2_@C/NG further fundamentally confer improved structural stability and reaction kinetics for lithium storage. As expected, the SnO_2_-SnS_2_@C/NG composite exhibited high reversible capacity (1201.2 mA h g^−1^ at the current rate of 0.1 A g^−1^), superior rate capability and exceptional long-life stability (944.3 mAh g^−1^ after 950 cycles at the current rate of 1.0 A g^−1^). The results demonstrate that the SnO_2_-SnS_2_@C/NG composite is a highly competitive anode material for LIBs.

## Introduction

In recent years, with the widespread use of portable electronic products and electric vehicles, lithium-ion battery technology needs to be rapidly upgraded (Armand and Tarascon, 2008; Wang et al., 2017a,b, 2019; Chen J. et al., 2018; Zheng et al., 2018; Tong et al., 2019). At present, the main problems faced by lithium-ion batteries (LIBs) are as follows: energy density, cycle life, safety and cost. These problems are mainly related to the anode and cathode of the battery. As far as anode materials are concerned, commercial graphite on the market today is unable to meet the increasing energy density requirements due to its lower capacity (about 372 mAh g^−1^) (Chen et al., 2018b; Xiao et al., 2018). In the past few decades, researchers developed many high-capacity and structurally stable anode materials, among which tin oxide anode materials with low discharge potential and natural abundance, received extensive attention and become a strong competitor for the next generation of lithium-ion battery anode materials (Chou et al., 2009; Sahoo and Ramaprabhu, 2018; Woo et al., 2018; Ye et al., 2019). Besides, owing to the conversion reaction (SnO_2_ + 4Li^+^ + 4e^−^ → Sn + 2Li_2_O) and alloying reaction (Sn + 4.4Li^+^ + 4.4e^−^ ↔ Li_4.4_Sn), SnO_2_ anodes possess a high theoretical capacity (1,494 mAh g^−1^) for lithium storage (Hu et al., 2017; Shao et al., 2017; Wang et al., 2018).

However, two main problems that delay the commercialization process of SnO_2_ anodes are poor conductivity and severe volume expansion. Researchers have done a lot of research work to solve these problems, and the most effective strategy at present is to combine with carbon materials (Du et al., 2014; Chen et al., 2018d; Hou et al., 2018; Li et al., 2018), which is indeed a great strategy. The introduction of carbon can limit the size of the material to obtain nanomaterials (Wang et al., 2013; Chen et al., 2018c). Nanoparticles can effectively reduce the absolute volume change of each particle, greatly improving the structural stability of the material. Furthermore, the charge-diffusion path of ions and electrons are greatly shortened and a large number of electrochemically active sites existed in nanoparticles (Ying and Han, 2017). On the other hand, the introduction of carbon can greatly improve the electrical conductivity of the material and enhance the structural stability of the material. Compared with other carbon materials, graphene has a two-dimensional layer structure with a single atomic thickness, remarkable structural flexibility, excellent electrical conductivity, high mechanical strength, and a large specific surface area. Therefore, graphene has obvious advantages in the application of electrode materials, and is an ideal substrate for dispersing and limiting active substances (Chen et al., 2018a; Li et al., 2019).

Recently, some researchers propose that the charge transfer kinetics can be enhanced by constructing a reasonable heterogeneous structure with built-in driving force (Jiang et al., 2017; Li et al., 2018; Ren et al., 2018). The synergistic effect of ion/electron transport originating from solid-solid heterojunctions is particularly important for electrode performance of essentially enhanced kinetics. In this context, superior electrochemical performance was demonstrated for SnS/SnO_2_ (Li et al., 2018), MoS_2_/SnS_2_ (Jiang et al., 2017), MnMoO_4_/CoMoO_4_ (Chen H. et al., 2014), and Fe_2_O_3_/Mn_2_O_3_ (Ren et al., 2018). SnS_2_ is a CdI2 type layered structure material with a large interlayer spacing (0.5899 nm) and a narrow band gap (Xu et al., 2015). Moreover, since the bond energy of Sn-S is weaker than the bond energy of Sn-O, this will promote the conversion of SnS_2_ to Sn (Zhang S. et al., 2017; Wu et al., 2019). Therefore, the conversion reaction kinetics can be greatly improved by constructing a heterostructure between SnO_2_ and SnS_2_. Whereas, the development of synergistic ultra-fine heterogeneous interface lithium storage materials still faces enormous challenges.

Herein, we propose a facile and scalable fabrication approach of heterostructured SnO_2_-SnS_2_@carbon/nitrogen-doped reduced graphene oxide (SnO_2_-SnS_2_@C/NG) composite. In this hierarchical structure, the as-prepared SnO_2_-SnS_2_ nanoparticles were intimately embedded in carbon matrix with nitrogen-doped graphene, constructing an intimate cross-link conductive framework. Apart from the heterojunction structure of SnO_2_-SnS_2_, the carbon matrix with nitrogen-doped graphene accommodates the volume variation and facilitates the transport of lithium ions during continuous lithation/delithation cycling. The SnO_2_-SnS_2_@C/NG composite with hierarchical structure can fundamentally possess enhanced kinetics and reaction reversibility, thereby ensuring the stable cycling life.

## Experimental Section

### Preparation of SnO_2_-SnS_2_@C/NG Composite

All chemical reagents in this experiment are of analytical grade. The preparation method of SnO_2_@C/reduced graphene oxide (SnO_2_@C/rGO) refers to our previous report (Li et al., 2019). Firstly, 30 mL of graphene oxide (GO) solution (0.5 mg mL^−1^) was added to the beaker, and then 0.25 g of SnCl_2_ and 0.15 g of sodium alginate were thoroughly dispersed in the above GO solution, and slowly added to 30 mL of deionized water to form uniform solution. Secondly, the resulting solution was then hydrothermally treated at 180°C for 12 h. After that, the obtained gray powder was calcined at 450°C for 1 h under Ar atmosphere to obtain SnO_2_@C/rGO composite. Finally, 0.2 g of the obtained precursor and 0.4 g of thioacetamide were added to 50 ml of deionized water, and hydrothermally treated at 200°C for 15 h to obtain the desired SnO_2_-SnS_2_@C/NG composite. SnO_2_-SnS_2_@C was prepared using the above method with 0.3 g of sodium alginate, and without using GO solution. SnO_2_@C was prepared using the above method with 0.3 g of sodium alginate, and without using GO solution and no further vulcanization.

### Characterization

The structure of SnO_2_-SnS_2_ composite was determined by X-ray diffraction (XRD, Bruker D8) with Cu Kα radiation. The degree of the disorder of carbon was detected by Raman spectroscopy (HORIBA, Jobin-Yvon Lab RAM Aramis). The form of various elements in the SnO_2_-SnS_2_@C/NG was characterized by X-ray photoelectron spectroscopy (XPS, K-Alpha). The macroscopic morphology of the composite was observed by scanning electron microscopy (SEM, Zeiss Gemini DSM 982) and the microscopic morphology of the composite was observed by transmission electron microscopy (TEM, FEI Tecnai, G2 F20 STWIN). Thermogravimetric analyses (TGA, NETZSCH STA 409PG/PC) of the composites were performed in air with a heating rate of 5°C min^−1^.

The electrochemical properties of the composites were evaluated using CR2025 cells. The composite material, conductive carbon (SP) and polyvinylidene fluoride (weight ratio 7:2:1) were dispersed in N-methylpyrrolidinone, and uniformly mixed to prepare a slurry. The slurry was uniformly coated on a copper foil current collector and dried at 120°C overnight, and the dried electrode sheets were controlled to have a loading density of 1.1–1.5 mg cm^−2^. The CR2025 batteries were assembled in a closed glove box, in which lithium tablets were used as the counter electrodes, and 1 M LiPF_6_ in ethylene carbonate and dimethyl carbonate (1:1, vol) was used as the electrolyte. The galvanostatic discharge/charging performances were tested by the battery test system (CT2001A, LAND) and the measured potential range was from 0.01 to 3.0 V. Cyclic voltammetry curves (CV, 0.01–3.0 V) and electrochemical impedance spectroscopy (EIS, 0.01–10^5^ Hz) were recorded by electrochemical workstation measurements (CHI1000C, CH Instruments).

## Results and Discussion

**Scheme 1:**
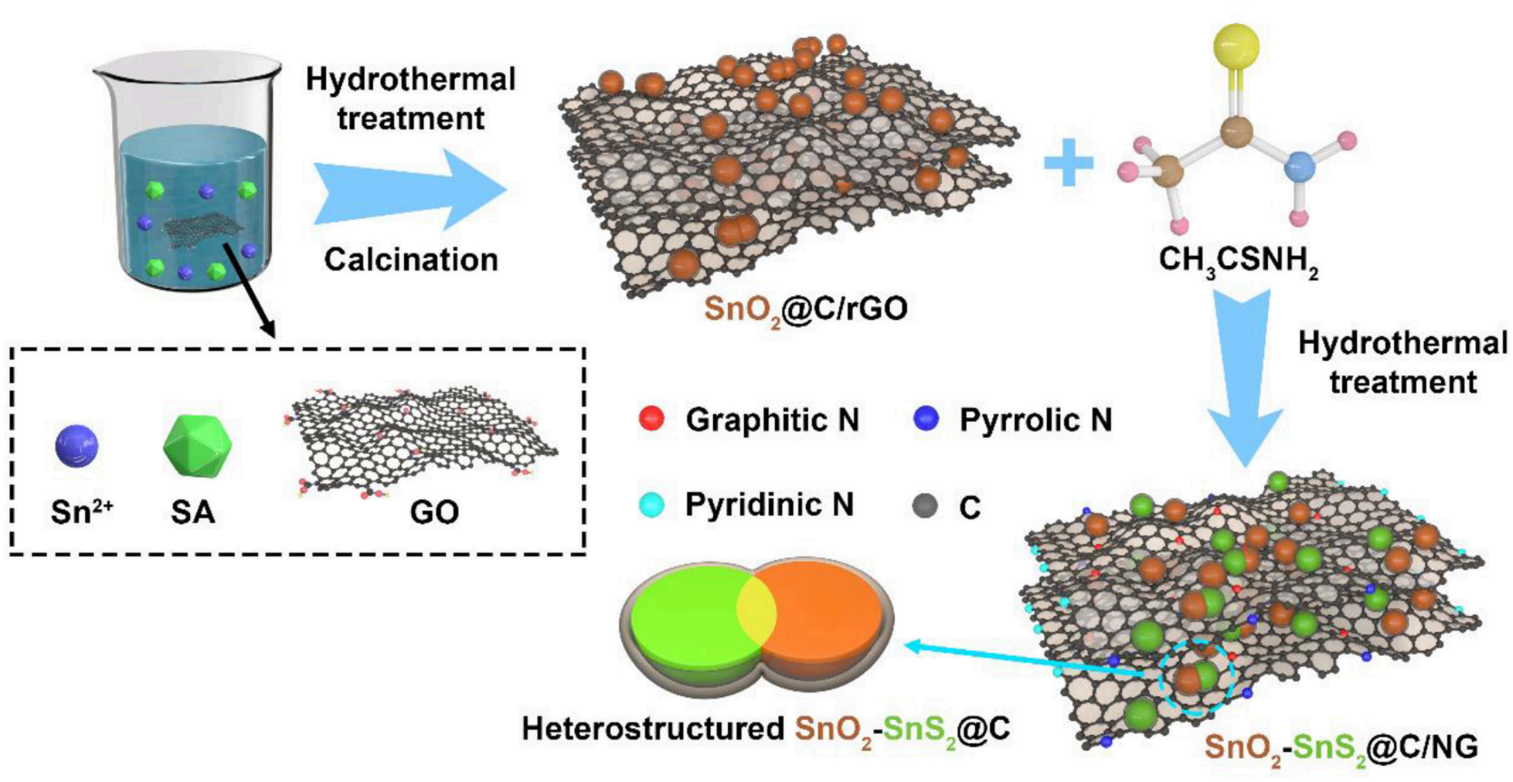
Schematic illustration of the preparation process of SnO_2_-SnS_2_@C/NG composite.

The representative fabrication process of heterostructured SnO_2_-SnS_2_@C/NG is presented in Scheme 1. Firstly, the SnO_2_@C/rGO composite was synthesized by hydrothermal method, using sodium alginate and GO to produce carbon matrix (C/rGO). Then, SnO_2_ was partially vulcanized and grapheme was doped by nitrogen under hydrothermal reaction to finally obtain SnO_2_-SnS_2_@C/NG composite with a heterostructure structure, in which the thioacetamide works as a vulcanizing agent and a nitrogen dopant.

**Figure 1:**
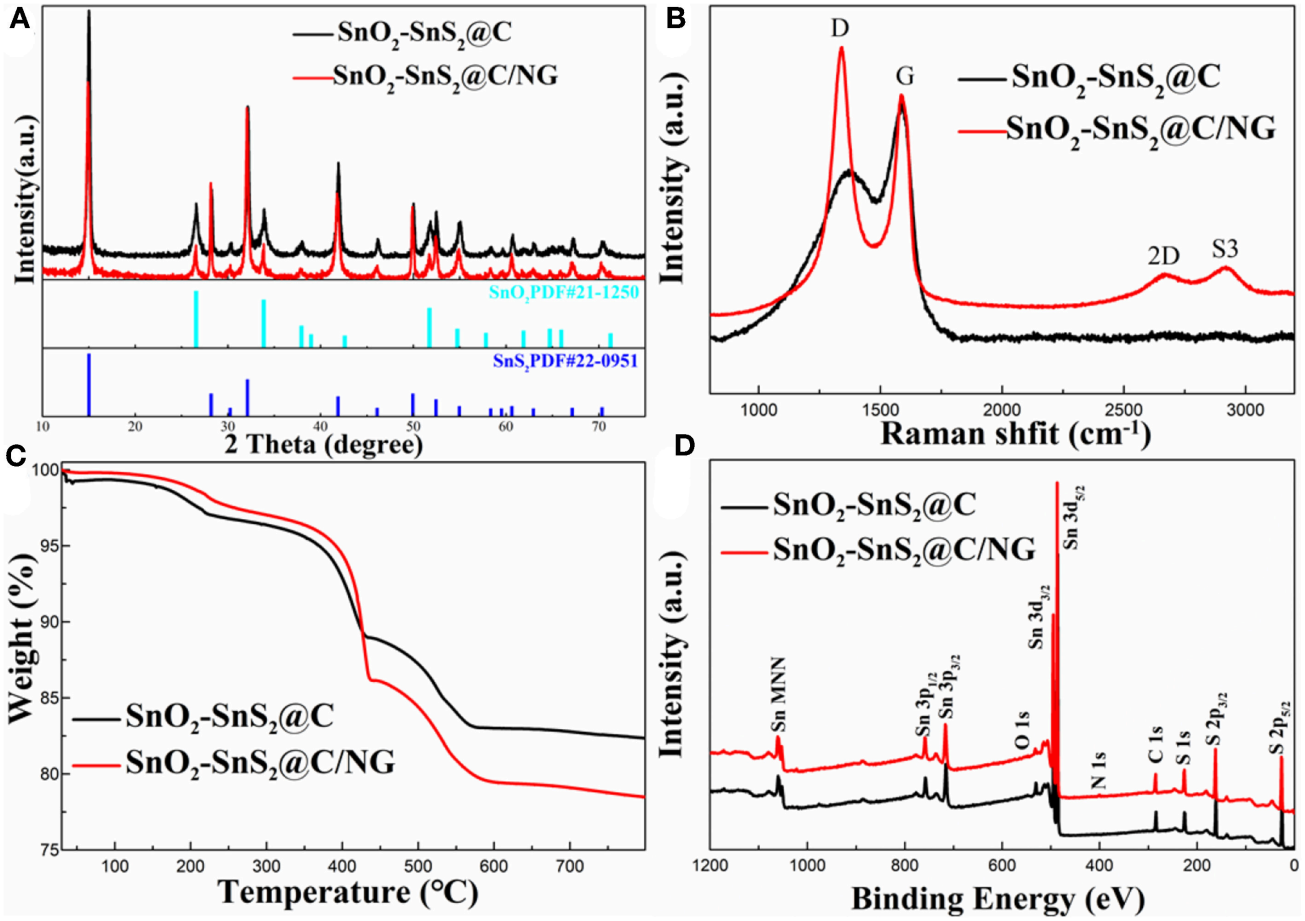
**(A)** XRD patterns, **(B)** Raman spectra, **(C)** TGA curves, and **(D)** XPS spectra of SnO_2_-SnS_2_@C and SnO_2_-SnS_2_@C/NG composites.

X-ray diffraction (XRD) patterns were collected to reveal the phase structures of the as-prepared SnO_2_-SnS_2_@C/NG and SnO_2_-SnS_2_@C composites. As exhibited in Figure 1A, the sharp diffraction peaks for both samples were consistent with SnO_2_ (PDF No#21-1250) and SnS_2_ (PDF#22-0951), respectively. The strong diffraction peaks represent a high degree of crystallinity of the composite. The results indicate that the composites consisted of SnO_2_ and SnS_2_ mixtures. Additionally, no obvious peaks indexed into carbon were observed, suggesting the amorphous state of carbon and graphene. SnO_2_@C sample (Figure S1A) was consistent with SnO_2_ (PDF No#88-0287). To demonstrate the existence of graphene in composites, Raman spectroscopy at the range of 800–3.200 cm^−1^ were collected in Figure 1B. It shows two obvious peaks at 1,337 and 1,583 cm^−1^, assigning to the disorder of carbon materials for D-band and characteristic of sp^2^ hybridized carbon for G-band (Liu et al., 2010; Zhang et al., 2016). Compared with the SnO_2_-SnS_2_@C and SnO_2_@C (Figure S1B) (I_D_/I_G_ = 0.72 and 0.71), the I_D_/I_G_ ratio in SnO_2_-SnS_2_@C/NG (1.20) was much higher, suggesting that the existence of graphene, which is beneficial for the rapid electron transport. In addition, it can be found in the spectra that the SnO_2_-SnS_2_@C/NG composite had two distinct peaks at 2670.3 and 2916.1 cm^−1^ due to the 2D and S3 bands, respectively (Liu et al., 2010). Notably, the symmetric 2D band indicates the presence of a single layer of graphene in the SnO_2_-SnS_2_@C/NG composite (Shah et al., 2017). To determine the carbon and SnS_2_ content, TGA was estimated in air, as displayed in Figure 1C. As can be seen from the figure, the weight loss can be divided into three stages, which are divided into the following: the first stage is room temperature to 250°C, the second stage is 250 to 450°C, and the third stage is 450 to 600°C. The weight loss in the first stage is due to the release of adsorbed water. The weight loss in the second stage corresponds to the conversion of SnS_2_ to SnO_2_ (SnS_2_+3O_2_ = SnO_2_+SO_2_↑) (Lu et al., 2018), meanwhile, SnO_2_-SnS_2_@C and SnO_2_-SnS_2_@C/NG composites lost weights of 8.04 and 11.05 wt%, respectively. It can be calculated that the contents of the SnS_2_ in SnO_2_-SnS_2_@C and SnO_2_-SnS_2_@C/NG composites material are 45.75 and 62.88 wt%, respectively, and the content of SnO_2_ produced from those are 37.71 and 51.83 wt%, respectively. The weight loss in the third stage is ascribed to the oxidation of carbon, resulting in weight losses of 5.85 and 6.83 wt% for the SnO_2_-SnS_2_@C and SnO_2_-SnS_2_@C/NG composites, respectively. Until the last stage, the remaining products were all SnO_2_, and the SnO_2_-SnS_2_@C and SnO_2_-SnS_2_@C/NG composites retain 82.98 and 79.39 wt% (This contains two parts of SnO_2_: one part is the original SnO_2_ in the composites and the other part is the SnO_2_ converted from SnS_2_), respectively. Furthermore, it is inferred the specific composition of each component in the composite, as listed in Table 1. Surface chemical elements of SnO_2_-SnS_2_@C and SnO_2_-SnS_2_@C/NG composites were characterized by XPS analyses (Figure 1D). The S, O, Sn, and C elements were found in both composites in the full spectrum of XPS. Compared with SnO_2_-SnS_2_@C, it can be observed that SnO_2_-SnS_2_@C/NG had a weak intensity peak of N 1s at 400 cm^−1^, suggesting that the N element has been doped into the graphene lattice.

**Table 1:** Contents of each component in the composites.

	**SnO_**2**_ (wt%)**	**SnS_**2**_ (wt%)**	**Carbon (wt%)**
SnO_2_@C (Figure S1C)	90.56	0	5.80
SnO_2_-SnS_2_@C	45.27	45.75	5.85
SnO_2_-SnS_2_@C/NG	62.88	27.56	6.83

**Figure 2:**
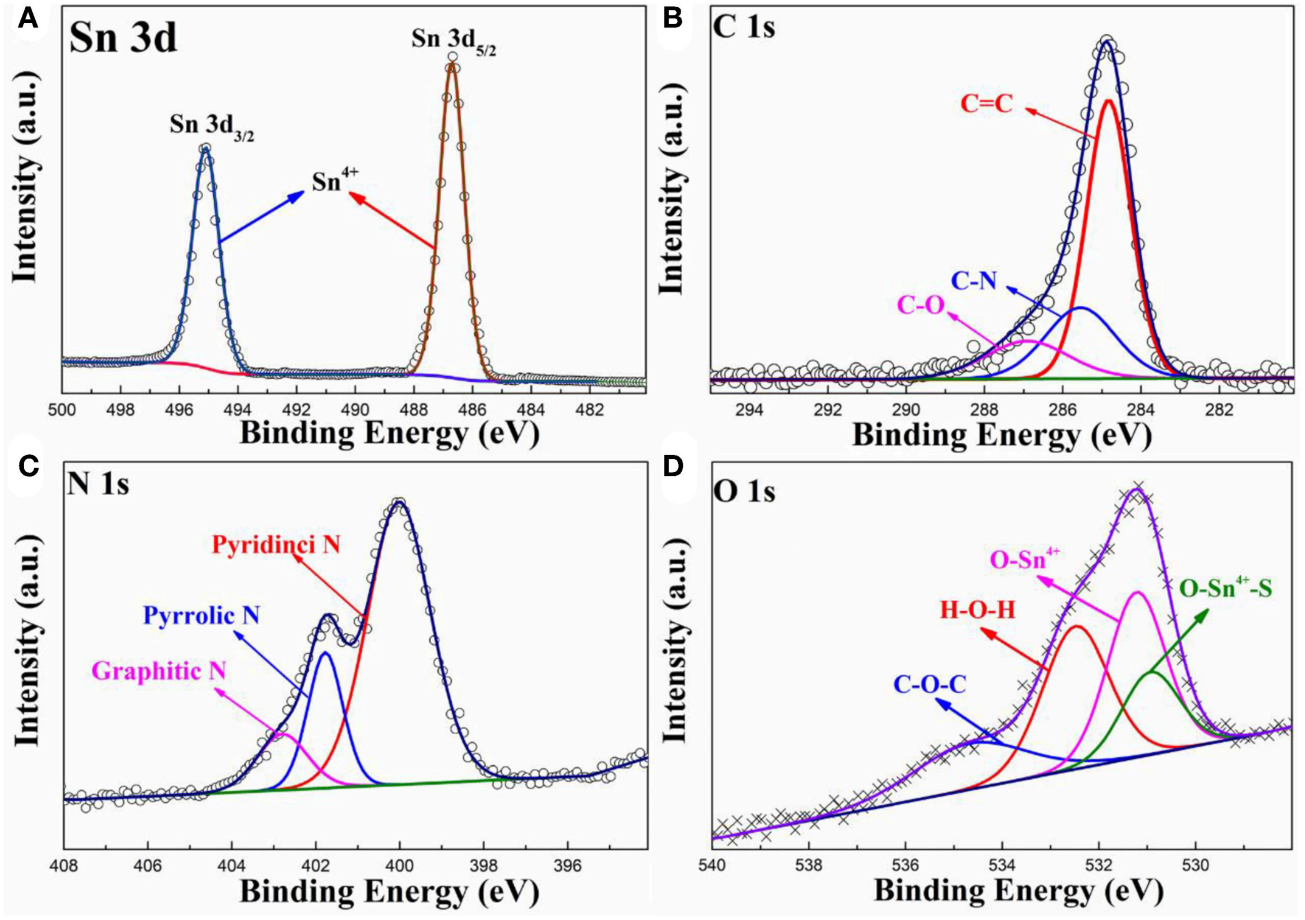
High-resolution XPS spectra of **(A)** Sn 3d, **(B)** C 1s, **(C)** N 1s, and **(D)** O 1s spectra for SnO_2_-SnS_2_@C/NG composite.

To obtain more information about the chemical state of the elements in the prepared SnO_2_-SnS_2_@C/NG composite, High-resolution XPS spectra are depicted in Figure 2. As shown in Figure 2A, the high-resolution Sn 3d XPS spectrum of the SnO_2_-SnS_2_@C/NG composite can be fitted to the intensity peaks at 487.2 and 495.6 eV, respectively, confirmed that tin in the composite exists in the form of Sn^4+^ (Lu et al., 2018). The C 1s spectrum is applied to further analyze the carbon form in the SnO_2_-SnS_2_@C/NG composite. As observed in Figure 2B, the high-resolution C 1s XPS spectrum of SnO_2_-SnS_2_@C/NG can be fitted to three peaks located at 284.7, 285.5, and 286.9 eV for C = C, C-N, and C-O, respectively (Li et al., 2018). The relative weak intensity of the C-O peak indicates that the oxide in the graphene is partially reduced. Besides, the presence of C-N peak further demonstrates nitrogen doping into the graphene lattice, which opens the band gap, adjusts the conductivity type, changes the electronic structure, and increases the free carrier density in the graphene, thereby improving the conductivity of SnO_2_-SnS_2_@C/NG electrodes. By fitting the high-resolution N 1s spectrum (Figure 2C), it can be found that N 1s spectrum can be divided into three peaks located in at 400.0, 401.8, and 402.7 eV, corresponding to the pyridinic, pyrrolic, and graphitic nitrogen atoms, respectively (Wu et al., 2018). Figure 2D shows the O 1s spectrum of the SnO_2_-SnS_2_@C/NG composite, which can be fitted into four types of oxygen-containing bond units located at 530.9, 531.2, 532.5, and 534.9 eV, respectively. The peaks at 530.9, 531.2 and 532.5 eV correspond to O-Sn^4+^, H-O-H, and C-O-C bonds, respectively. Notably, the peak at 534.9 eV corresponds to the S-Sn^4+^-O bond, which is a strong evidence of the presence of a heterojunction in the composite (Zhang et al., 2018). This confirmed the presence of a chemical bond between SnO_2_ and SnS_2_ particles.

**Figure 3:**
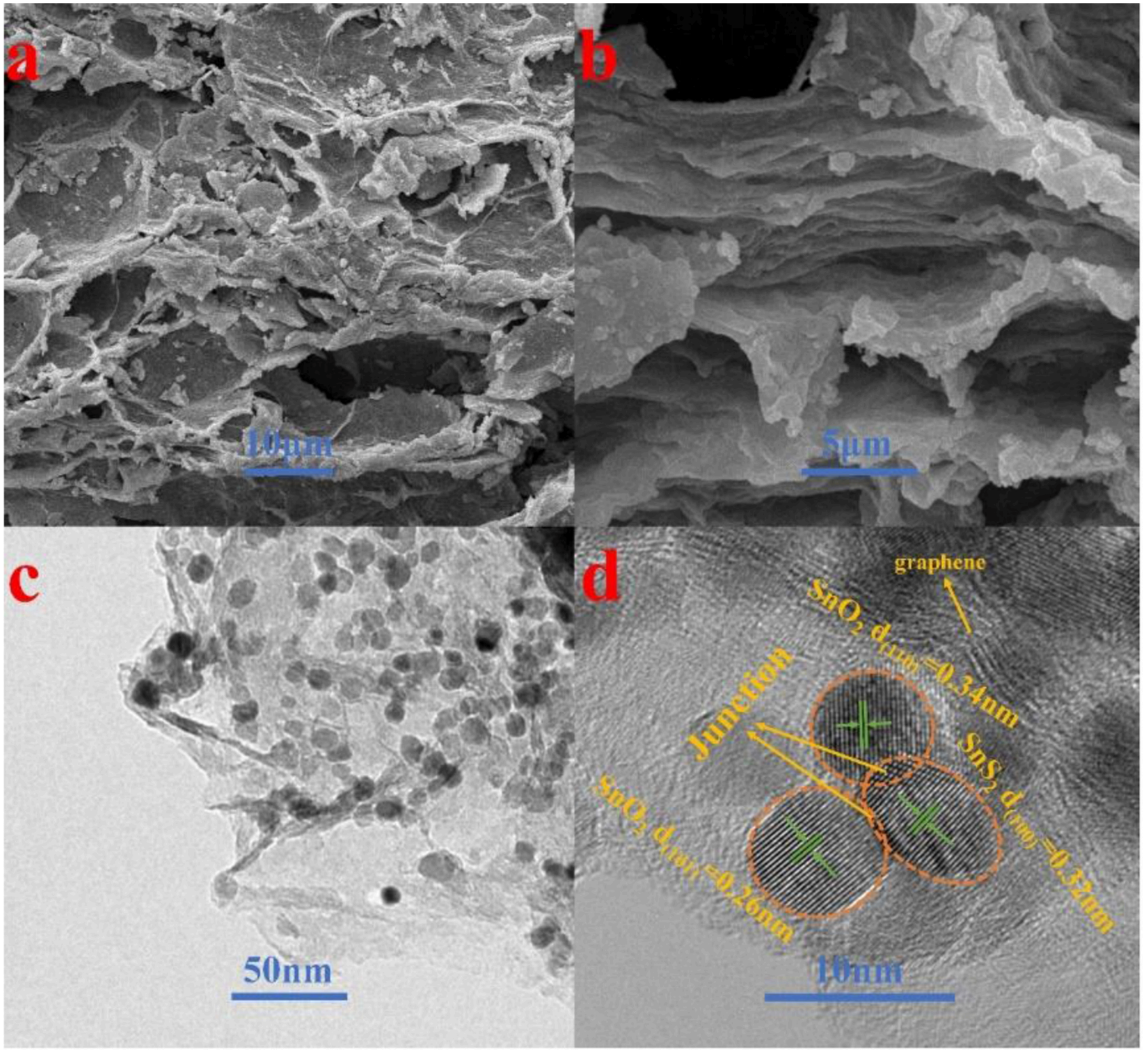
**(a,b)** SEM, **(c)** TEM, and **(d)** HRTEM images of SnO_2_-SnS_2_@C/NG composite.

**Figure 4:**
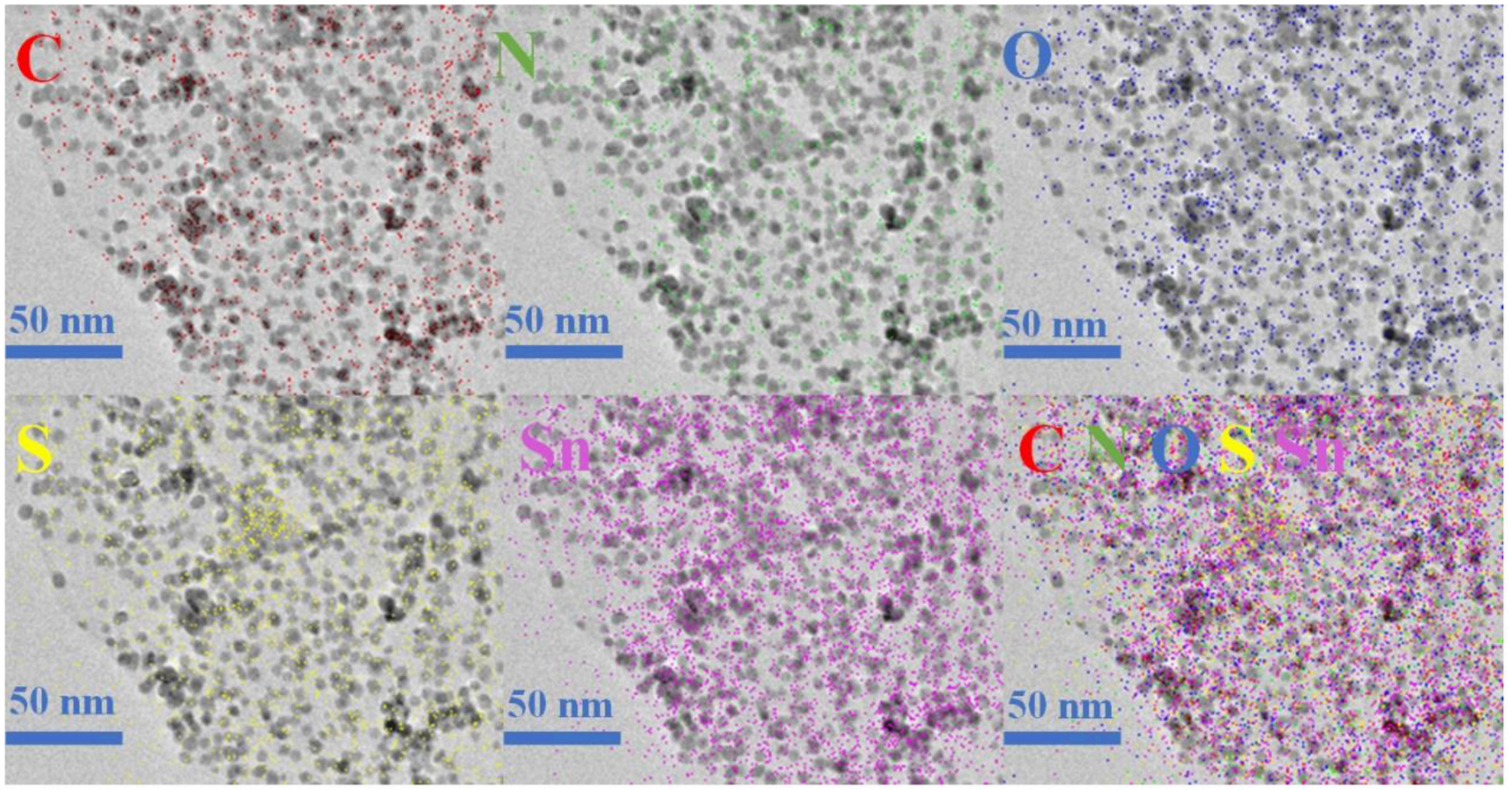
EDS mapping images of SnO_2_-SnS_2_@C/NG composite.

The morphology and detailed structural feature of the prepared composite were observed by SEM, TEM and HRTEM. As can be observed in Figure 3a, the morphology of the SnO_2_-SnS_2_@C/NG composite shows an orderly lamination structure, forming a fluffy gap between the layers, which is very beneficial to the infiltration of the electrolyte. The ultrafine SnO_2_-SnS_2_ nanoparticles embedded into the crumpled carbon matrix, thereby forming tremendous voids in the composite. Due to the uniform distribution of nanosized particles, which are hardly observed from the SEM image (Figure 3b). In the absence of graphene-involved combination, the particles of the SnO_2_-SnS_2_@C composite were agglomerated into microspheres of 200–300 μm diameter (Figure S2A). TEM is also applied to characterize the detailed structural feature of SnO_2_-SnS_2_@C/NG composite. As presented in Figure 3c, it can be clearly observed that the nanoparticles are perfectly wrapped by a single layer of graphene sheet. The graphene sheet in the figure also has void spaces and wrinkles, which can significantly suppress the volume expansion of the nanoparticles and enhance the conductivity of the composites. Under the observation of TEM, it is found that the microspheres of SnO_2_-SnS_2_@C composite (Figure S2B) are composed of primary particles of about 100–500 nm, and the surface of the primary particles is covered with an amorphous carbon. Figure 3d clearly depicts the nanoparticles with the diameter of 5–10 nm and high degree of crystallization, which are uniformly embedded in graphene with a curved lattice fringe of 0.424 nm. The particle in nanometer size can significantly shorten the lithium diffusion path and greatly improve the rate performance of the composite. Meanwhile, Figure 3d also displays the obvious several small nanocrystals with lattice spacings of 0.26, 0.34 and 0.32 nm, corresponding to (101) and (110) planes of SnO_2_ and (100) plane of SnS_2_, respectively (Shi and Lu, 2014; Chen et al., 2015; Shan et al., 2015; Yin et al., 2017). More importantly, as denoted in Figure 3d, the heterojunctions are formed in the overlapped junction regions. In Figure S2C, the heterojunction phenomenon of SnO_2_-SnS_2_@C can also be found. The formation of heterojunctions composed of (101) plane of SnO_2_, (100) plane of SnS_2_, and (110) plane of SnO_2_, which strongly confirms the existence of heterogeneous SnO_2_-SnS_2_. Such a hierarchical structure is imperative for circumventing the self-aggregation of Sn-based nanoparticles, as well as the Sn and/or Li_x_Sn nanoparticles produced during cycling. Additionally, EDS mapping illustrates the homogeneous distribution of C, N, O, S, and Sn elements on the SnO_2_-SnS_2_@C/NG composite, as presented in Figure 4, which is consistent with the TEM analysis. The characterization of various physical and chemical means suggests that SnO_2_-SnS_2_@C/NG composite is a well-designed hierarchical structure, in which heterojunctions composed of SnO_2_ and SnS_2_ ultrafine nanoparticles are encapsulated in nitrogen-doped graphene sheets.

**Figure 5:**
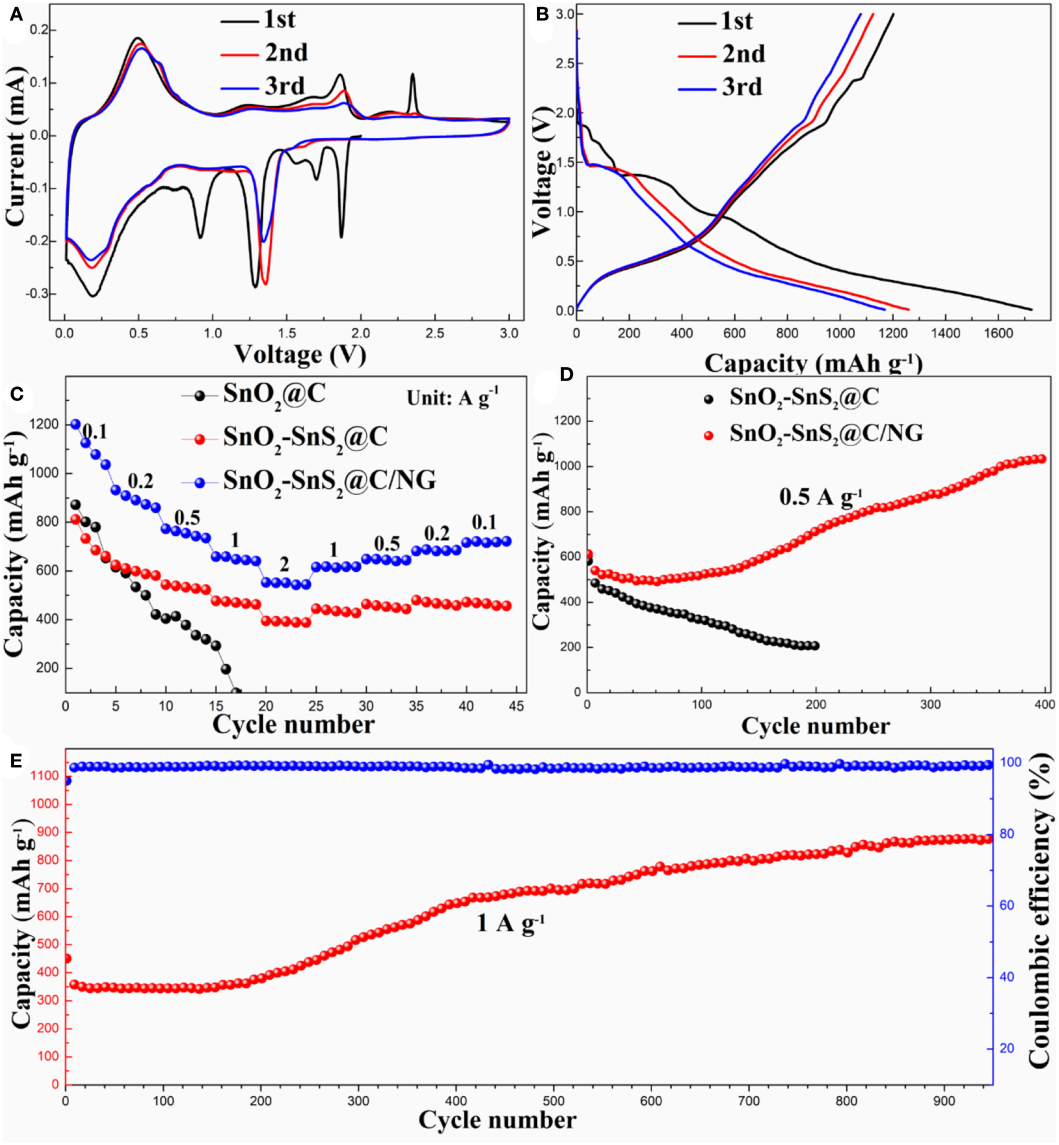
**(A)** CV profiles and **(B)** the charge/discharge curves in the initial three cycles for SnO_2_-SnS_2_@C/NG composite. **(C)** Rate capabilities and **(D)** cycle performances for SnO_2_-SnS_2_@C and SnO_2_-SnS_2_@C/NG composites at current rate of 0.5 A g^−1^, and **(E)** long cycling property for SnO_2_-SnS_2_@C/NG composite at current rate of 1 A g^−1^.

In order to explore the mechanism reaction of the composite during the charging and discharging process, the CV curve in the first three cycles of SnO_2_-SnS_2_@C/NG electrode was studied, as shown in Figure 5A. During the first scan of the cathodic process, the peaks at 1.87 and 1.70 V are attributed to the Li deintercalation in the SnS_2_ layer, and there was no phase decomposition in this process, so the corresponding peaks were not found in the subsequent cycle (Yin et al., 2017). Decomposition of SnS_2_ to metallic tin and Li_2_S results in a peak at 1.29 V. The conversion of SnO_2_ and SnO to Sn produced a cathode peak at around 0.92 V and formed a synchronous products of Li_2_O and SEI film (Chen et al., 2015). The strong cathode peak at 0.18 V is attributed to Li_x_Sn produced by the alloying reaction of elemental tin and lithium ions, and peak at the same potential was also observed in subsequent cycles, indicating that the alloying reaction here is reversible. In the anodic scan process, the strong peak at 0.5 V is caused by the de-alloying of Li_x_Sn, corresponding to the 0.18 V peak in the cathodic scan. The peak at 1.86 V is attributed to the oxidation and sulfurization of Sn to SnO_2_ and SnS_2_, respectively. The peak at 2.35 V is related to partial decomposition of Li_2_S, which also produces the first small irreversible capacity (Lu et al., 2018). It is worth noting that due to phase decomposition and structural collapse, the cathodic peak of 1.29 V in the first cycle shifted to 1.36 V in the subsequent cycle (Lu et al., 2018). Meanwhile, Figure 5B shows the first three discharge/charge curves of the SnO_2_-SnS_2_@C/NG composite, with a voltage window of 0.01–3.0 V and a current rate of 0.1 A g^−1^, which is consistent with the CV results. The first discharge/charge capacity achieved 1727.2 and 1201.2 mAh g^−1^, respectively, with initial coulombic efficiency of 69.54%. The excess capacity may come from the formation of SEI film on the surface of active materials, lithium-insertion reaction in acetylene black and interfacial storage, which is similar to the phenomena of other Sn-based anodes (Chen Y. et al., 2014; Fu et al., 2018; Li et al., 2019). The low initial coulombic efficiency is mainly due to the formation of SEI films, which is usually observed for the nanosized anode materials (Liu et al., 2014; Yang et al., 2018), whereas the coulombic efficiency approached 99.9% in the following cycles. In contrast, the initial charge capacity for SnO_2_-SnS_2_@C reached 812.4 mAh g^−1^ under the same condition (Figure S3), which is relatively lower than that of SnO_2_-SnS_2_@C/NG.

As depicted in Figure 5C, the rate capabilities of the composite electrodes were performed at various current rates ranging from 0.1 to 2 A g^−1^. The SnO_2_-SnS_2_@C electrodes delivered stable capacities of 815.7, 628.6, 544.5, 478.6, and 395.8 mAh g^−1^ at the rates of 0.1, 0.2, 0.5, 1, and 2 A g^−1^, respectively. When current rate returned to 0.1 A g^−1^, it recovered to 475.7 mAh g^−1^. In contrast, the rate performance of the SnO_2_@C electrode was relatively poorer, as its capacity dropped sharply when the current density increased until the cell failed. The possible reason is that the heterojunction in the SnO_2_-SnS_2_@C composite can significantly improve the conductivity, resulting in an increase in rate performance. Apparently, the rate performance of SnO_2_-SnS_2_@C/NG composite was the best. The SnO_2_-SnS_2_@C/NG electrodes delivered stable capacities of 1206.7, 936.3, 777.3, 666.3, and 559.0 mAh g^−1^ at the rates of 0.1, 0.2, 0.5, 1, and 2 A g^−1^, respectively. When the current rate returns to 0.1 A g^−1^, it still can recover to 724.1 mAh g^−1^. The SnO_2_-SnS_2_@C/NG composite also exhibited the best cycle performance. The capacity of SnO_2_-SnS_2_@C/NG composite electrode at current rate of 0.5 A g^−1^ reached 717.6 mAh g^−1^ after 200 cycles and gradually increased to 1039.4 mAh g^−1^ after 400 cycles (Figure 5D). In addition, the charge and discharge curves for the 400th cycle of the SnO_2_-SnS_2_@C/NG composite are shown in the Figure S4. The capacity ratio in the voltage of 2.0–3.0 V was about 42%, which may be related to the reversible formation of the polymer gel-like film. However, the SnO_2_-SnS_2_@C electrode exhibited a low capacity of 208.0 mA h g^−1^ at 0.5 A g^−1^ after 200 cycles. Remarkably, at the high current rate of 1 A g^−1^ (Figure 5E), the SnO_2_-SnS_2_@C/NG electrode still performed well after 950 cycles, exhibiting a strong reversible capacity of 944.3 mAh g^−1^, with the stabilized coulombic efficiency of around 99.5% throughout the whole cycling, confirming the merits of SnO_2_-SnS_2_@C/NG composite during the long-term cycling. In contrast with previous reported anodes materials, the SnO_2_-SnS_2_@C/NG composite exhibits outstanding cycle stability at higher current rates and exhibits outstanding electrochemical performance (as shown in Table S1). It is noteworthy that the capacity had a slightly decrease during the first 50 cycles, then gradually increased during the subsequent cycles, and finally maintaining a stable performance. This phenomenon is attributed to the electrode activation and reversible formation of the polymer gel-like film, followed by the continuous reconstruction of SEI film on the surface of nanomaterials (Wang et al., 2013; Zhou et al., 2013; Liu et al., 2014).

**Figure 6:**
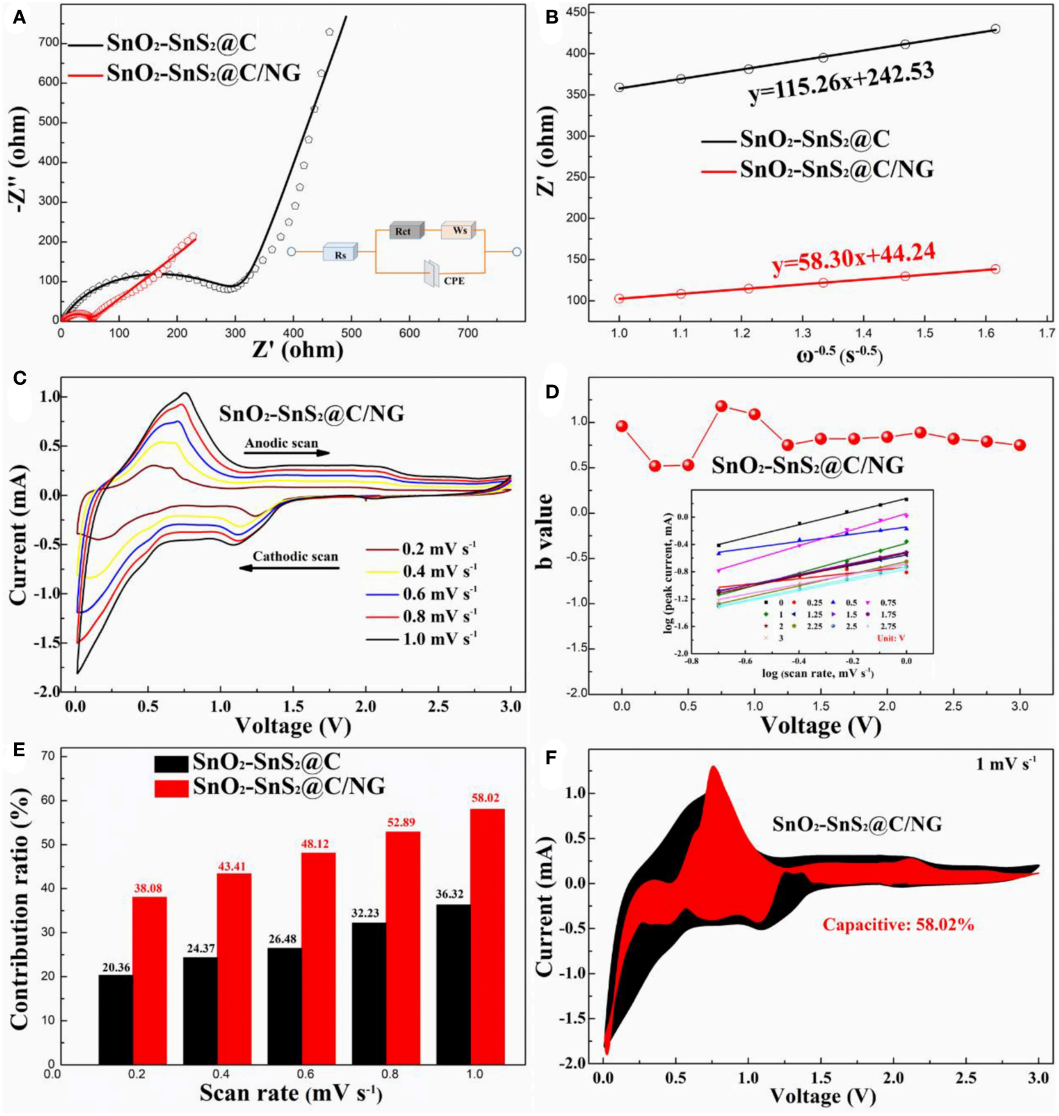
**(A)** Nyquist plots and corresponding fitted curves of SnO_2_-SnS_2_@C and SnO_2_-SnS_2_@C/NG electrodes before cycles (the inset is the modified equivalent circuit); **(B)** Nyquist plots and corresponding relationship between the real resistance (Z') and angular frequency (ω^−0.5^) for SnO_2_-SnS_2_@C and SnO_2_-SnS_2_@C/NG electrodes; **(C)** CV curves of SnO_2_-SnS_2_@C/NG electrode at different scan rates from 0.2–1.0 mV s^−1^; **(D)** b values plotted against cell voltage of SnO_2_-SnS_2_@C/NG electrode for cathodic scans; **(E)** the percentage of the capacitance contribution for SnO_2_-SnS_2_@C and SnO_2_-SnS_2_@C/NG at different scan rates; **(F)** CV curve of SnO_2_-SnS_2_@C/NG at a sweep rate of 1.0 mV s^−1^, and the estimated capacitive current contribution is shown in the shaded region.

In order to gain a deep understanding of the excellent electrochemical properties and to evaluate the lithium storage kinetics and charge transfer capacity of the composites, the SnO_2_-SnS_2_@C and SnO_2_-SnS_2_@C/NG electrodes were investigated by EIS. As displayed in Figure 6A, all Nyquist plots consist of a depressed semicircle at high frequency and a straight drift at low-medium frequency, which can be perfect fitted with an equivalent circuit as displayed in the insert of Figure 6A. Compared with charge-transfer resistance (R_ct_) of SnO_2_-SnS_2_@C (323.6 Ω), the R_ct_ of SnO_2_-SnS_2_@C/NG (47.37 Ω) is much smaller. Furthermore, the lithium-ion diffusion coefficient (D_Li+_) can be further calculated according to the following equations (Yi et al., 2017; Zhang B. et al., 2017; Tong et al., 2018) (R, gas constant; T, absolute temperature; A, surface area; n, average reacted electrons; F, Faraday constant; C, Li^+^ concentration, respectively):

(1) $${\text{D}}_{\text{Li}}^{+}={\text{R}}^{2}{\text{T}}^{2}{/2\text{A}}^{2}{\text{n}}^{4}{\text{F}}^{4}{\text{C}}^{2}\sigma^{2}$$

(2) $$\begin{array}{r} {\text{Z}}^{\prime }={\text{R}}_{\text{s}}+{\text{R}}_{\text{ct}}+\sigma \omega^{1/2} \end{array}$$

in which, σ can be calculated by the plot of Z' vs. ω^−0.5^ (angular frequency) as depicted in Figure 6B. Therefore, the calculated values of ${\text{D}}_{\text{Li}}^{+}$ listed in Table S2 imply the rapid diffusion of lithium ions and excellent reaction kinetics of the SnO_2_-SnS_2_@C/NG electrode.

In order to further explore the excellent rate performance of the SnO_2_-SnS_2_@C/NG electrode, CV analyses at various scan rates from 0.2 to 1 mV s^−1^ were carried out (Figure 6C). Considering that SnO_2_-SnS_2_@C/NG is superior than SnO_2_-SnS_2_@C in high-rate performance and fast lithium ion transport, the pseudocapacitance contribution of SnO_2_-SnS_2_@C/NG may be more than that of SnO_2_-SnS_2_@C during charge/discharge process. In this case, the ratios of capacitive contribution can be further quantitatively quantified by separating the current response (*i*) at a fixed potential (V) into capacitive contribution (*k*_1_*v*) and diffusion-controlled contribution (*k*_2_*v*^1/2^), according to the following equation:

(3) $$\begin{array}{r} i=k_{1}v+k_{2}v^{1/2}=av^{b} \end{array}$$

in which, *a* and *b* present adjustable values. It must be noted that there is no proportional relationship between the measured current (*i*) and the square root of the scan rate (*v*) at a certain potential, suggesting the redox process can hardly be assigned to entire ion-diffusion control. The value of *b* is usually between 0.5 and 1, where 0.5 represents the diffusion control process and 1.0 illustrates the ideal pseudocapacitive behavior according to previous mechanism (Zhao et al., 2017). As displayed in Figure 6D, by fitting log(*i*) and log(*v*) at various redox potentials, several b values >0.8 can be obtained therefrom, demonstrating a dominated pseudocapacitive-controlled contribution process for the SnO_2_-SnS_2_@C/NG electrode. Furthermore, the contribution of total pseudocapacitive at a specific scan rate can be accurately calculated by the following equation ((Zhao et al., 2017); Zhang et al., 2018):

(4) $$\begin{array}{r} i\left(V\right)/v^{1/2}=k_{1}v^{1/2}+k_{2} \end{array}$$

By carefully comparing the capacity contributions at different scan rates (Figure 6E), it is not difficult to conclude that the percentage of pseudocapacitive contribution increases gradually as the scan rate increases, and the lithiation/delithiation reaction of the SnO_2_-SnS_2_@C/NG composite gradually becomes capacitive control, and the pseudocapacitive-controlled contribution ratios of the SnO_2_-SnS_2_@C/NG composite are always higher than those of the SnO_2_-SnS_2_@C composite. As highlighted in Figure 6F, when the scan rate is 1 mV s^−1^, nearly 58.02% of the total current (red region) is attributed to the pseudocapacitive contribution for the SnO_2_-SnS_2_@C/NG electrode, which is the maximum at all scan rates.

The distinct pseudocapacitive behavior and enhanced reaction kinetic proved by EIS results are originated from the synergistic effect of SnO_2_-SnS_2_ heterojunction incorporated into the highly conductive N-doped graphene. The ultrafine SnO_2_-SnS_2_ nanoparticles can provide shorten transport distance of lithium ions, especially the formation of heterojunctions at the interface of SnO_2_-SnS_2_ nanoparticles effectively enhance the electronic transmission, which greatly promote the rate capability. Furthermore, nitrogen-doped graphene also plays a key role in lithium ion and electronic conductivity, which can build highly-conductive framework among the dispersive nanoparticles with integrity architecture, efficiently facilitating the lithium transport path and thus resulting in the excellent electrochemical performance.

**Figure 7:**
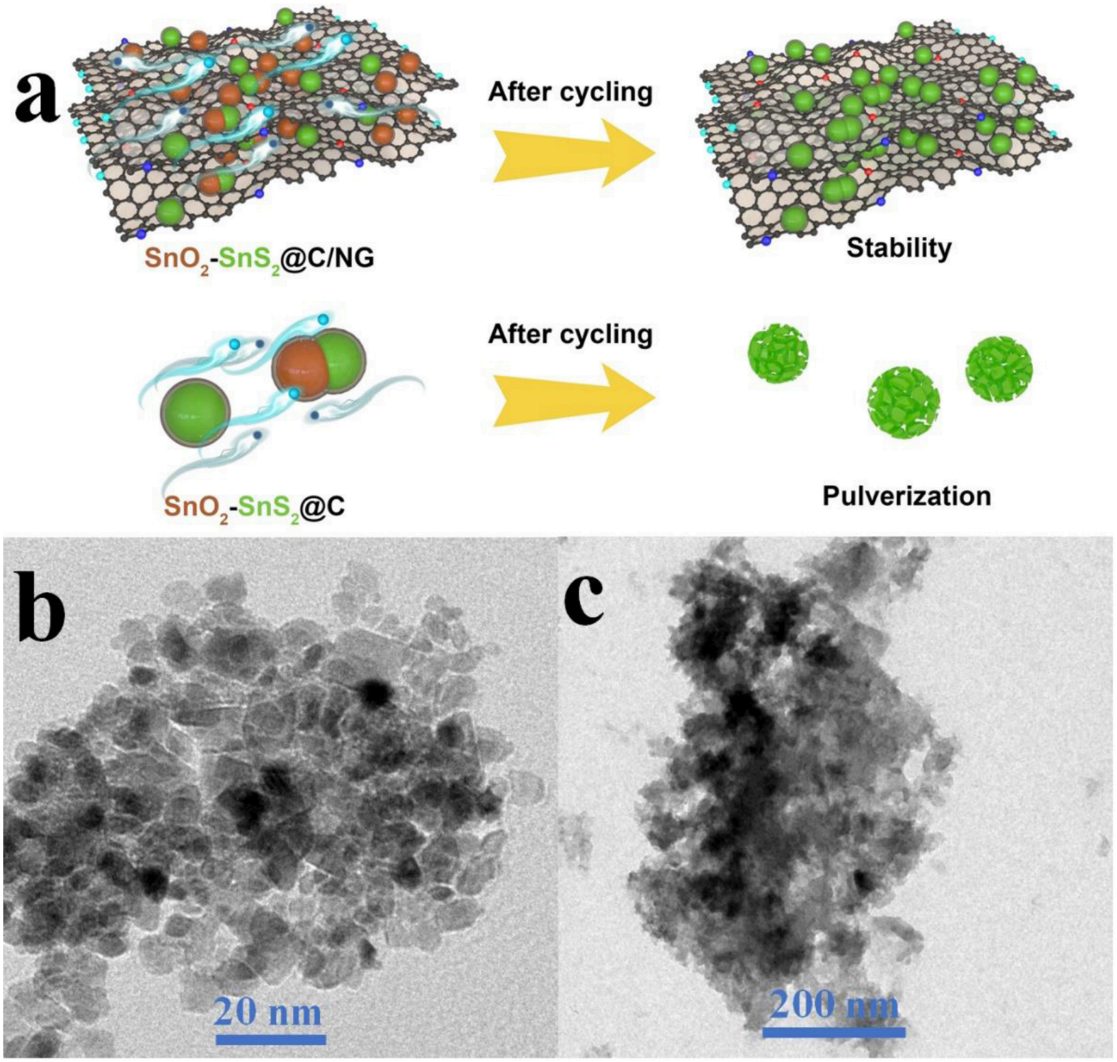
**(a)** Schematic illustration of the robust structure for SnO_2_-SnS_2_@C/NG and SnO_2_-SnS_2_@C composites. TEM images of **(b)** SnO_2_-SnS_2_@C/NG and **(c)** SnO_2_-SnS_2_@C composites after cycling test.

The nitrogen-doped graphene nanosheets interconnect the nanoparticles and act as cushions for its volume expansion during long-term lithiation/delithiation process, further enhancing the structural stability and improving the life-cycle of entire electrode, as schematically illustrated in Figure 7a. The correlations between cycling properties and structural features for the as-prepared SnO_2_-SnS_2_@C/NG and SnO_2_-SnS_2_@C composites were characterized by TEM tests after 400th cycle at the current rate of 0.5 A g^−1^. As presented in Figure 7b, the flake morphology of SnO_2_-SnS_2_@C/NG was well-maintained after long-term cycling. The repeated volume expansion is effectively restricted by the robust architecture of the carbon matrix, in which the amorphous carbon locks the SnO_2_-SnS_2_ nanoparticles on the nitrogen-doped graphene sheets, inhibiting its severe volume variation. In comparison, SnO_2_-SnS_2_@C composite without incorporation of graphene suffer from the particle pulverization and agglomeration after continuous cycling (Figure 7c), and the nanoparticles are easily detached from the current collector, which leads to the unstable structure and severe electrode degradation with bad cycling performance. Furthermore, the repeated formation of thick SEI films overlying on the surface SnO_2_-SnS_2_ particles has adverse effects on the reversibility of conversion reaction, directly impeding the lithium ion and electron transfer and resulting in the continuous capacity decrease. All results indicate that heterostructured SnO_2_-SnS_2_@C/NG composite demonstrates enhanced structural stability and superior electrochemical performance.

## Conclusions

In this work, the well-designed SnO_2_-SnS_2_@C/NG composite was ingeniously fabricated by a facile approach, in which the heterojunction of SnO_2_-SnS_2_ was created and embedded into the carbon matrix with highly conductive nitrogen-doped graphene nanosheets. As expected, SnO_2_-SnS_2_@C/NG composite exhibited a high reversible capacity (1201.2 mA h g^−1^ at current rate of 0.1 A g^−1^), superior rate capability and long-life stability (1039.4 mAh g^−1^ after 400 cycles at current rate of 0.5 A g^−1^, and 944.3 mAh g^−1^ after 950 cycles at current rate of 1.0 A g^−1^). The excellent electrochemical properties may be ascribed to the following reasons. The lithium ion diffusion path is shortened in SnO_2_-SnS_2_ nanoparticles with ultrafine size, and reversibility and reaction kinetics are enhanced through the introduction of heterostructure. Meanwhile, the nitrogen-doped graphene sheets improve the electronic conductivity and structural stability, which can effectively accommodate the volume variation and maintain the steady formation of SEI films without particle pulverization. This strategy contributes a new insight for preparing the high-performance electrode materials for LIBs.

## Data Availability

The raw data supporting the conclusions of this manuscript will be made available by the authors, without undue reservation, to any qualified researcher.

## Author Contributions

HL carried out the experiment and wrote the manuscript. XW, JZ, and TA participated in the experiment. BZ, ZD and WY contributed to the discussion. HT supervised the experiment and proofread the manuscript.

### Conflict of Interest Statement

The authors declare that the research was conducted in the absence of any commercial or financial relationships that could be construed as a potential conflict of interest.
