# Supplementary material for: Heterostructured SnO2-SnS2@C Embedded in Nitrogen-Doped Graphene as a Robust Anode Material for Lithium-Ion Batteries
Source: Front Chem. 2019 May 14;7:339. doi: 10.3389/fchem.2019.00339 (PMC6527815; doi:10.3389/fchem.2019.00339)
Supplement: Supplementary file 1 [file Data_Sheet_1.docx]

**Supporting information**

**
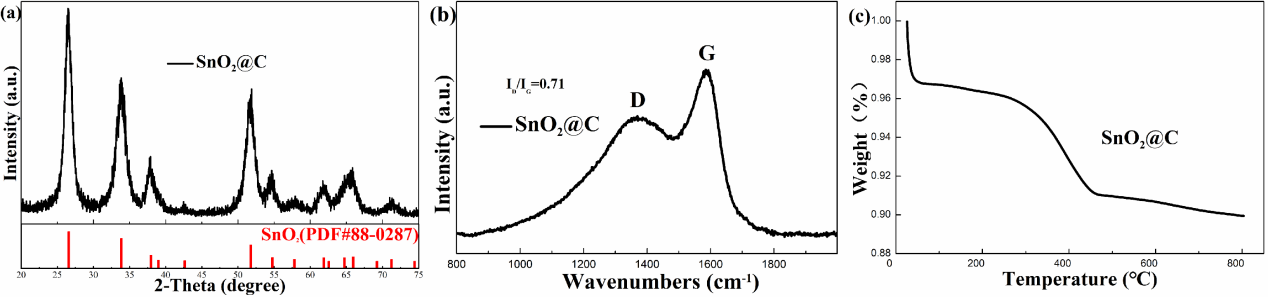
**

**FIGURE S1** (a) XRD pattern, (b) Raman spectrum, and (c) TGA curve of SnO_2_@C composite.


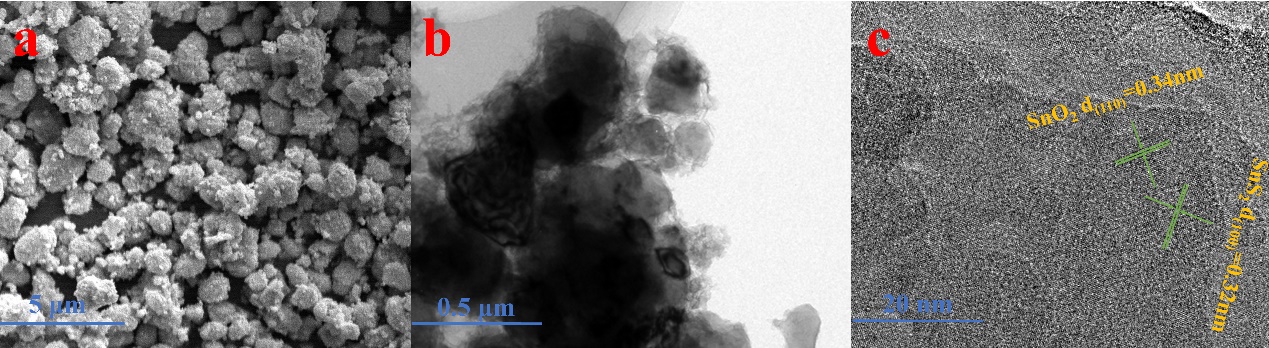


**FIGURE S2** (a) SEM, (b) TEM, and (c) HRTEM images of SnO_2_-SnS_2_@C composite.





**FIGURE S3** The charge/discharge curves of SnO_2_-SnS_2_@C electrode in the initial three cycles.

**
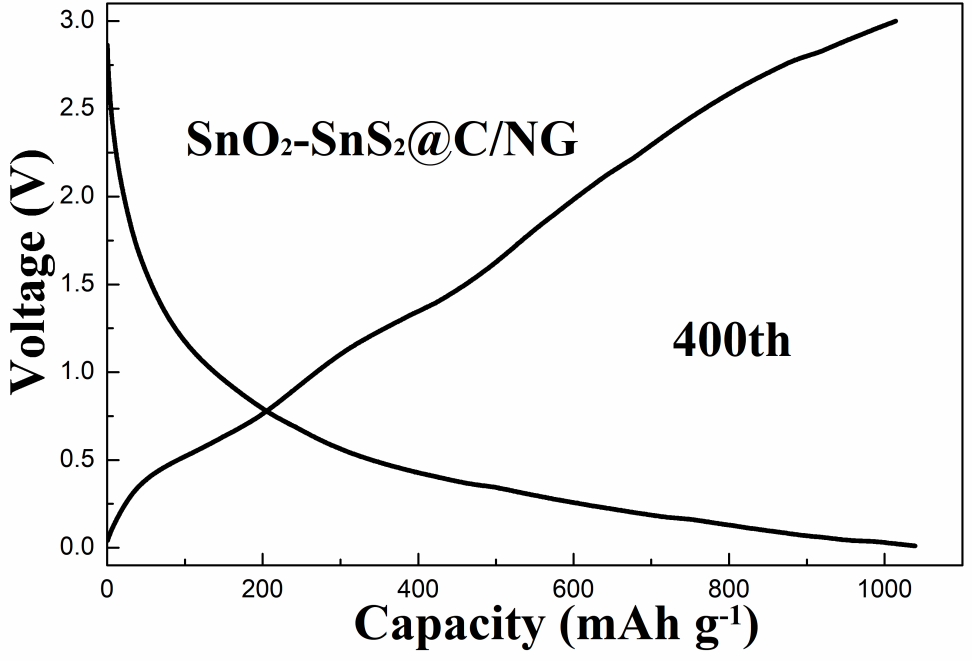
**

**FIGURE S4** The charge and discharge curves for the 400th cycle of SnO_2_-SnS_2_@C/NG electrode**.**

**TABLE S1** Comparison of SnO_2_-SnS_2_@C/NG composite with other Sn-based anode materials and their electrochemical performances.

| Electrodes | Current densities  (A g^−1^) | Capacities  (mAh g^−1^) | Cycles | References |
| --- | --- | --- | --- | --- |
| Sphere-like SnS_2_/SnO_2_ | 0.1 | 588.15 | 50 | [S1] |
| Echinus-like SnO_2_@SnS_2_ Nanospheres | 0.1 | 548 | 100 | [S2] |
| SnS_2_/SnO_2_/C | 0.1 | 712 | 300 | [S3] |
| C@SnS-SnO_2_@NGr | 0.8  1 | 880  630 | 200  1000 | [S4] |
| C@NG@ SnS-SnO_2_ | 0.1 | 1236 | 110 | [S5] |
| C@SnS-SnO_2_ on carbon nanofibers | 1 | 827 | 500 | [S6] |
| SnO_2_@SnS_2_@rGO Hollow Spheres | 0.2  0.5 | 583  487 | 100  100 | [S7] |
| Sn/SnO/NGNSs | 1 | 853 | 250 | [S8] |
| Sn/SnO_x_@C | 0.2 | 1001 | 240 | [S9] |
| **SnO_2_-SnS_2_@C/NG** | **0.5**  **1** | **1039.4**  **944.3** | **400**  **950** | **Current study** |

**TABLE S2** The fitting results of kinetic parameters for SnO_2_-SnS_2_@C and SnO_2_-SnS_2_@C/NG electrodes.

| Electrodes | R_s_(Ω) | R_ct_(Ω) | σ | D_Li_（cm^2^ s^-1^） |
| --- | --- | --- | --- | --- |
| SnO_2_-SnS_2_@C | 10.32 | 323.60 | 115.26 | 9.52×10^-15^ |
| SnO_2_-SnS_2_@C/NG | 3.32 | 47.37 | 58.30 | 7.25×10^-13^ |

**REFERENCES**

[S1] Yin, L., Chai, S., Huang, J., Kong, X., Pan, L. (2017). Preparation of hierarchical SnS_2_/SnO_2_ anode with enhanced electrochemical performances for lithium-ion battery. *Electrochim. Acta, 238*, 168-177. doi:10.1016/j.electacta.2017.03.183

[S2] Wei, S., Lu, B. (2014). Nanoscale kirkendall effect synthesis of echinus-like SnO_2_ @SnS_2_ nanospheres as high performance anode material for lithium ion batteries. *Electrochim. Acta, 133*(7), 247-253. doi:10.1016/j.electacta.2014.04.013

[S3] Chen, C. Y., Yokoshima, T., Nara, H., Momma, T., Osaka, T. (2015). One-step hydrothermal synthesis of SnS_2_/SnO_2_/C hierarchical heterostructures for Li-ion batteries anode with superior rate capabilities. *Electrochim. Acta, 183*, 78-84. doi:10.1016/j.electacta.2015.05.079

[S4] Li, M., Deng, Q., Wang, J., Jiang, K., Shang, L., Hu, Z., Chu, J. (2018). In-situ gas reduction in reversible SnS-SnO_2_@N-doped graphene anodes for high-rate and lasting lithium storage. *J. Alloy. Compd., 769*, 1007-1018. doi:10.1016/j.jallcom.2018.08.038

[S5] Shan, J., Liu, Y., Liu, P., Huang, Y., Su, Y., Wu, D., Feng, X. (2015). Nitrogen-doped carbon-encapsulated SnO_2_–SnS/graphene sheets with improved anodic performance in lithium ion batteries. *J. Mater. Chem. A 3*(47), 24148-24154. doi:10.1039/C5TA06617D

[S6] Lian, Q., Zhou, G., Zeng, X., Wu, C., Wei, Y., Cui, C.,Li, C. C. (2016). Carbon coated SnS/SnO_2_ heterostructures wrapping on CNFs as an improved-performance anode for Li-ion batteries: lithiation-induced structural optimization upon cycling. *ACS Appl. Mater. Interfaces, 8*(44),30256-30263. doi:10.1021/acsami.6b10391.

[S7] Xu W.W., Xie Z.Q., Cui X.D., Zhao K.N , Zhang L., Dietrich G., Kerry M D., Wang Y. (2015). Hierarchical graphene-encapsulated hollow SnO_2_@SnS_2_ nanostructures with enhanced lithium storage capability. *ACS Appl. Mater. Interfaces, 7*(40), 22533-22541. doi:10.1021/acsami.5b06765

[S8] Du, F.H., Liu, Y.S., Long, J., Zhu, Q.C., Wang, K.X., Wei, X., Chen, J.S. (2014). Incorporation of heterostructured Sn/SnO nanoparticles in crumpled nitrogen-doped graphene nanosheets for application as anodes in lithium-ion batteries. *Chem. Commun., 50*(69), 9961-9964. doi:10.1039/c4cc04187a

[S9] Yang, J., Chen, S., Tang, J., Tian, H., Tao, B., Zhou, X. (2018). Rod-like hierarchical Sn/SnO_x_@C nanostructures with enhanced lithium storage properties. *Appl. Sur. Sci., 435*, 203-209. doi:10.1016/j.apsusc.2017.11.085
